# 2-Amino­pyrimidinium 4-hy­droxy­pyridinium-2,6-dicarboxyl­ate monohydrate

**DOI:** 10.1107/S1600536810029533

**Published:** 2010-08-04

**Authors:** Hossein Eshtiagh-Hosseini, Milad Mahjoobizadeh, Masoud Mirzaei

**Affiliations:** a Department of Chemistry, School of Sciences, Ferdowsi University of Mashhad, Mashhad 917791436, Iran

## Abstract

In the crystal structure of the title compound, C_4_H_6_N_3_^+^·C_7_H_4_NO_5_^−^·H_2_O, inter­molecular N—H⋯N, N—H⋯O and O—H⋯O hydrogen bonds link the cations and anions into almost planar sheets parallel to (102). These hydrogen-bonded sheets are packed into the crystal with the formation of centrosymmetric voids of 68 Å^3^, which are filled by the water mol­ecules, each of which is disordered over four positions.

## Related literature

For related structures, see: Aghabozorg *et al.* (2008 ▶); Moghimi *et al.* (2005 ▶); Hall *et al.* (2000 ▶); Lynch & Jones (2004 ▶); Eshtiagh-Hosseini *et al.* (2010 ▶); Smith *et al.* (2006*a* ▶,*b* ▶). For hydrogen bonding, see: Desiraju (1989 ▶).
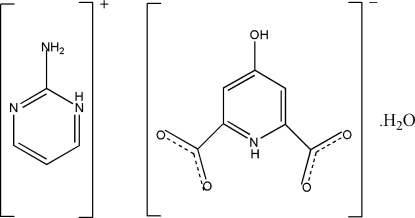


## Experimental

### 

#### Crystal data

C_4_H_6_N_3_^+^·C_7_H_4_NO_5_^−^·H_2_O
*M* *_r_* = 296.25
Monoclinic, 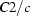

*a* = 17.822 (2) Å
*b* = 12.2233 (14) Å
*c* = 12.0676 (14) Å
β = 103.345 (2)°
*V* = 2557.8 (5) Å^3^
*Z* = 8
Mo *K*α radiation
μ = 0.13 mm^−1^
*T* = 100 K
0.20 × 0.20 × 0.15 mm

#### Data collection

Bruker SMART APEXII CCD area detector diffractometer
Absorption correction: multi-scan (*SADABS*; Bruker, 2005 ▶) *T*_min_ = 0.970, *T*_max_ = 0.983
14881 measured reflections
3383 independent reflections
2703 reflections with *I* > 2/s(*I*)
*R*_int_ = 0.031

#### Refinement

*R*[*F* ^2^ > 2σ(*F* ^2^)] = 0.046
*wR*(*F* ^2^) = 0.134
*S* = 0.90
3383 reflections
203 parameters
13 restraints
H-atom parameters constrained
Δρ_max_ = 0.45 e Å^−3^
Δρ_min_ = −0.33 e Å^−3^

### 

Data collection: *APEX2* (Bruker, 2005 ▶); cell refinement: *SAINT* (Bruker, 2005 ▶); data reduction: *SAINT*; program(s) used to solve structure: *SHELXTL* (Sheldrick, 2008 ▶); program(s) used to refine structure: *SHELXTL*; molecular graphics: *SHELXTL*; software used to prepare material for publication: *publCIF* (Westrip, 2010 ▶).

## Supplementary Material

Crystal structure: contains datablocks I, global. DOI: 10.1107/S1600536810029533/cv2745sup1.cif

Structure factors: contains datablocks I. DOI: 10.1107/S1600536810029533/cv2745Isup2.hkl

Additional supplementary materials:  crystallographic information; 3D view; checkCIF report

## Figures and Tables

**Table 1:** Hydrogen-bond geometry (Å, °)

*D*—H⋯*A*	*D*—H	H⋯*A*	*D*⋯*A*	*D*—H⋯*A*
N4—H4*NA*⋯O2	0.91	1.90	2.795 (2)	172
N4—H4*NB*⋯N2^i^	0.89	2.11	2.990 (2)	171
N3—H3*N*⋯O1	0.93	1.76	2.683 (2)	171
O3—H3*O*⋯O4^ii^	0.95	1.55	2.4910 (19)	171

Symmetry codes: (i) 
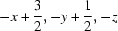
; (ii) 
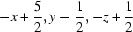
.

**Fig. 1.**
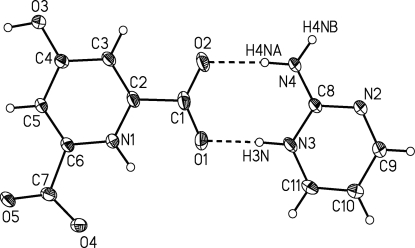
View of the title compound with the atomic numbering and 50% probability displacement ellipsoids. Dashed lines denote hydrogen bonds. The disordered water molecules were omitted for clarity.

## supplementary crystallographic information

### Comment

A number of cases were reported in which a proton transferred from a carboxylic acid to an amine to form some novel proton transfer compounds (Aghabozorg *et al.*, 2008). There have been several attempts to prepare proton transfer compounds involving carboxylic acids and amines, for example, ion pairs have been reported between H_2_pyzdc and various organic bases such as 8-hydroxy quinoline (Smith *et al.*, 2006*a*), guanidine (Smith *et al.*, 2006*b*) and 2,4,6-triamine-1,3,5-triazin (Eshtiagh-Hosseini *et al.*, 2010). However, there are few papers only concerning the 4-hydroxypyridine-2,6-dicarboxylic acid (hereafter hypydcH_3_). For example, ion pair including guanidine (Moghimi *et al.*, 2005) and hydrated form of hypydcH_3 _(Hall *et al.*, 2000) have been reported. In this paper, we have chosen hypydcH_3_ and 2-aminopyromidine (hearafter 2-apym) to obtain an ionic molecular crystal.

The crystal structure of the title proton transfer compound shows that a single proton from one of the carboxyl groups was transferred to the N-ring atom of the 2-apym molecule (Fig. 1). On the other hand, an interesting feature exhibited by the crystal structure is that an intramolecular proton transfer has occurred from the other carboxyl group to the N atom of the aromatic ring of hypydcH_3_. The cation is hydrogen bonded to the anion with a cyclic *R*_2_^2^(8) pattern (Fig. 1) in similar manner as reported by Lynch (Lynch & Jones, 2004). In the crystal structure, intermolecular N—H···N, N—H···O and O—H···O hydrogen bonds (Table 1) link cations and anions into almost planar sheets parallel to the (102) plane. These hydrogen-bonded sheets are further packed into crystal with the formation of centrosymmetric voids of 68 Å^3^, which are filled by the water molecules disordered between four positions each.

### Experimental

The title proton transfer compound was synthesized *via* the reaction of hypydcH_3_ (0.01 g, 0.5 mmol) with 2-apym (0.01 g, 0.1 mmol) in a aqueous solution (25 ml). The solution was stirred for 3 h in 358 K, and finally a colourless solution was obtained. Prism colourless crystals were obtained after slow evaporation of the solvent at RT.

### Refinement

The solvate water molecule was disordered over four positions near the inversion center with the occupancies refined to 0.292 (3), 0.249 (3), 0.236 (3) and 0.224 (3), respectively. The O(water)-bound hydrogen atoms were positioned manually with O—H 0.85-0.88 Å. The hydroxy and amino H atoms were found in a difference Fourier map. C-bound H atoms were positioned geometrically. All hydrogen atoms were refined as riding,  with *U*_iso_(H) = 1.2 - 1.5 *U*_eq_ of the parent atom.

### Crystal data

**Table:**

C_4_H_6_N_3_^+^·C_7_H_4_NO_5_^−^·H_2_O	*F*(000) = 1232
*M**_r_* = 296.25	*D*_x_ = 1.539 Mg m^−^^3^
Monoclinic, *C*2/*c*	Mo *K*α radiation, λ = 0.71073 Å
Hall symbol: -C 2yc	Cell parameters from 2135 reflections
*a* = 17.822 (2) Å	θ = 3–30°
*b* = 12.2233 (14) Å	µ = 0.13 mm^−^^1^
*c* = 12.0676 (14) Å	*T* = 100 K
β = 103.345 (2)°	Prism, colourless
*V* = 2557.8 (5) Å^3^	0.20 × 0.20 × 0.15 mm
*Z* = 8	

### Data collection

**Table:**

Bruker SMART APEXII CCD area detector diffractometer	3383 independent reflections
Radiation source: fine-focus sealed tube	2703 reflections with *I* > 2/s(*I*)
graphite	*R*_int_ = 0.031
phi and ω scans	θ_max_ = 29.0°, θ_min_ = 2.0°
Absorption correction: multi-scan (*SADABS*; Bruker, 2005)	*h* = −24→24
*T*_min_ = 0.970, *T*_max_ = 0.983	*k* = −16→16
14881 measured reflections	*l* = −16→16

### Refinement

**Table:**

Refinement on *F*^2^	Primary atom site location: structure-invariant direct methods
Least-squares matrix: full	Secondary atom site location: difference Fourier map
*R*[*F*^2^ > 2σ(*F*^2^)] = 0.046	Hydrogen site location: mixed
*wR*(*F*^2^) = 0.134	H-atom parameters constrained
*S* = 0.90	*w* = 1/[σ^2^(*F*_o_^2^) + (0.0847*P*)^2^ + 2.9446*P*] where *P* = (*F*_o_^2^ + 2*F*_c_^2^)/3
3383 reflections	(Δ/σ)_max_ = 0.002
203 parameters	Δρ_max_ = 0.45 e Å^−^^3^
13 restraints	Δρ_min_ = −0.33 e Å^−^^3^

### Special details

**Table:**

Geometry. All e.s.d.'s (except the e.s.d. in the dihedral angle between two l.s. planes) are estimated using the full covariance matrix. The cell e.s.d.'s are taken into account individually in the estimation of e.s.d.'s in distances, angles and torsion angles; correlations between e.s.d.'s in cell parameters are only used when they are defined by crystal symmetry. An approximate (isotropic) treatment of cell e.s.d.'s is used for estimating e.s.d.'s involving l.s. planes.
Refinement. Refinement of *F*^2^ against ALL reflections. The weighted *R*-factor *wR* and goodness of fit *S* are based on *F*^2^, conventional *R*-factors *R* are based on *F*, with *F* set to zero for negative *F*^2^. The threshold expression of *F*^2^ > σ(*F*^2^) is used only for calculating *R*-factors(gt) *etc*. and is not relevant to the choice of reflections for refinement. *R*-factors based on *F*^2^ are statistically about twice as large as those based on *F*, and *R*- factors based on ALL data will be even larger.

### Fractional atomic coordinates and isotropic or equivalent isotropic displacement parameters (Å^2^)

**Table:**

	*x*	*y*	*z*	*U*_iso_*/*U*_eq_	Occ. (<1)
N1	1.14962 (8)	−0.04289 (11)	0.20255 (13)	0.0224 (3)	
H1N	1.1618	0.0308	0.2020	0.027*	
O1	1.04750 (7)	0.11863 (10)	0.14699 (12)	0.0282 (3)	
O2	0.94910 (7)	−0.00108 (11)	0.09859 (11)	0.0285 (3)	
O3	1.08809 (7)	−0.36262 (10)	0.17357 (11)	0.0255 (3)	
H3O	1.1310	−0.4104	0.1904	0.031*	
O4	1.29127 (7)	0.02724 (11)	0.27479 (14)	0.0350 (4)	
O5	1.33747 (7)	−0.14137 (11)	0.32416 (14)	0.0373 (4)	
C1	1.01849 (10)	0.02443 (14)	0.13234 (15)	0.0239 (3)	
C2	1.07491 (9)	−0.06993 (14)	0.16221 (14)	0.0208 (3)	
C3	1.05349 (9)	−0.17748 (14)	0.15300 (13)	0.0197 (3)	
H3A	1.0009	−0.1967	0.1244	0.024*	
C4	1.10958 (9)	−0.25973 (13)	0.18601 (14)	0.0197 (3)	
C5	1.18672 (9)	−0.22748 (13)	0.23042 (14)	0.0207 (3)	
H5A	1.2257	−0.2810	0.2551	0.025*	
C6	1.20497 (9)	−0.11862 (13)	0.23764 (15)	0.0222 (3)	
C7	1.28608 (10)	−0.07533 (15)	0.28376 (17)	0.0290 (4)	
N2	0.82237 (8)	0.35778 (12)	0.04751 (13)	0.0223 (3)	
N3	0.94887 (8)	0.28765 (12)	0.10759 (12)	0.0206 (3)	
H3N	0.9807	0.2273	0.1274	0.025*	
N4	0.84352 (8)	0.17156 (12)	0.06968 (13)	0.0233 (3)	
H4NA	0.8755	0.1131	0.0843	0.028*	
H4NB	0.7931	0.1639	0.0425	0.028*	
C8	0.87116 (9)	0.27213 (13)	0.07514 (14)	0.0194 (3)	
C9	0.85244 (10)	0.45737 (14)	0.05743 (15)	0.0244 (4)	
H9A	0.8184	0.5180	0.0396	0.029*	
C10	0.93171 (10)	0.47828 (14)	0.09281 (15)	0.0247 (4)	
H10A	0.9515	0.5508	0.1004	0.030*	
C11	0.97917 (9)	0.38928 (15)	0.11582 (14)	0.0227 (3)	
H11A	1.0335	0.3990	0.1376	0.027*	
O1W	0.8160 (2)	0.8577 (4)	−0.0224 (4)	0.0257 (5)	0.292 (3)
H1WA	0.8468	0.8115	0.0198	0.031*	0.292 (3)
H1WB	0.8055	0.9096	0.0223	0.031*	0.292 (3)
O2W	0.8104 (3)	0.9291 (4)	0.0129 (4)	0.0257 (5)	0.249 (3)
H2WA	0.8544	0.9496	0.0513	0.031*	0.249 (3)
H2WB	0.8178	0.8757	−0.0286	0.031*	0.249 (3)
O3W	0.8024 (3)	0.7046 (4)	0.0112 (5)	0.0257 (5)	0.236 (3)
H3WA	0.8188	0.7058	−0.0496	0.031*	0.236 (3)
H3WB	0.7571	0.6781	−0.0054	0.031*	0.236 (3)
O4W	0.8561 (3)	0.7918 (5)	0.0081 (5)	0.0257 (5)	0.224 (3)
H4WA	0.8132	0.8099	−0.0354	0.031*	0.224 (3)
H4WB	0.8853	0.8478	0.0157	0.031*	0.224 (3)

### Atomic displacement parameters (Å^2^)

**Table:**

	*U*^11^	*U*^22^	*U*^33^	*U*^12^	*U*^13^	*U*^23^
N1	0.0147 (6)	0.0179 (6)	0.0343 (8)	0.0014 (5)	0.0046 (5)	−0.0010 (5)
O1	0.0217 (6)	0.0226 (6)	0.0404 (7)	0.0071 (5)	0.0073 (5)	0.0056 (5)
O2	0.0163 (6)	0.0317 (7)	0.0376 (7)	0.0079 (5)	0.0064 (5)	0.0096 (5)
O3	0.0164 (5)	0.0187 (6)	0.0386 (7)	−0.0016 (4)	0.0010 (5)	0.0032 (5)
O4	0.0188 (6)	0.0212 (6)	0.0616 (9)	−0.0020 (5)	0.0024 (6)	−0.0111 (6)
O5	0.0145 (6)	0.0261 (7)	0.0649 (10)	0.0030 (5)	−0.0041 (6)	−0.0122 (6)
C1	0.0192 (8)	0.0243 (8)	0.0292 (8)	0.0072 (6)	0.0077 (6)	0.0066 (6)
C2	0.0144 (7)	0.0233 (8)	0.0245 (8)	0.0042 (6)	0.0041 (6)	0.0023 (6)
C3	0.0127 (7)	0.0233 (8)	0.0223 (7)	0.0006 (6)	0.0027 (5)	0.0014 (6)
C4	0.0156 (7)	0.0211 (7)	0.0218 (7)	−0.0011 (6)	0.0031 (6)	0.0014 (6)
C5	0.0139 (7)	0.0195 (7)	0.0270 (8)	0.0024 (5)	0.0010 (6)	−0.0010 (6)
C6	0.0132 (7)	0.0208 (7)	0.0311 (8)	0.0021 (6)	0.0018 (6)	−0.0039 (6)
C7	0.0151 (7)	0.0234 (8)	0.0460 (11)	−0.0010 (6)	0.0020 (7)	−0.0125 (7)
N2	0.0139 (6)	0.0213 (7)	0.0301 (7)	0.0010 (5)	0.0018 (5)	0.0020 (5)
N3	0.0112 (6)	0.0252 (7)	0.0235 (7)	0.0014 (5)	0.0001 (5)	0.0016 (5)
N4	0.0119 (6)	0.0206 (7)	0.0351 (8)	0.0015 (5)	0.0008 (5)	0.0035 (6)
C8	0.0128 (7)	0.0220 (7)	0.0220 (7)	0.0010 (5)	0.0015 (6)	0.0017 (6)
C9	0.0179 (8)	0.0225 (8)	0.0314 (9)	0.0018 (6)	0.0025 (6)	0.0023 (6)
C10	0.0189 (8)	0.0244 (8)	0.0297 (8)	−0.0034 (6)	0.0031 (6)	0.0002 (6)
C11	0.0139 (7)	0.0298 (8)	0.0231 (8)	−0.0037 (6)	0.0015 (6)	0.0003 (6)
O1W	0.0201 (11)	0.0265 (12)	0.0296 (13)	−0.0109 (9)	0.0042 (9)	−0.0058 (10)
O2W	0.0201 (11)	0.0265 (12)	0.0296 (13)	−0.0109 (9)	0.0042 (9)	−0.0058 (10)
O3W	0.0201 (11)	0.0265 (12)	0.0296 (13)	−0.0109 (9)	0.0042 (9)	−0.0058 (10)
O4W	0.0201 (11)	0.0265 (12)	0.0296 (13)	−0.0109 (9)	0.0042 (9)	−0.0058 (10)

### Geometric parameters (Å, °)

**Table:**

N1—C6	1.348 (2)	N3—C11	1.349 (2)
N1—C2	1.349 (2)	N3—C8	1.363 (2)
N1—H1N	0.9263	N3—H3N	0.9273
O1—C1	1.258 (2)	N4—C8	1.320 (2)
O2—C1	1.249 (2)	N4—H4NA	0.9056
O3—C4	1.3131 (19)	N4—H4NB	0.8882
O3—H3O	0.9466	C9—C10	1.402 (2)
O4—C7	1.264 (2)	C9—H9A	0.9500
O5—C7	1.234 (2)	C10—C11	1.367 (2)
C1—C2	1.518 (2)	C10—H10A	0.9500
C2—C3	1.366 (2)	C11—H11A	0.9500
C3—C4	1.409 (2)	O1W—H1WA	0.8664
C3—H3A	0.9500	O1W—H1WB	0.8805
C4—C5	1.411 (2)	O2W—H2WA	0.8508
C5—C6	1.368 (2)	O2W—H2WB	0.8509
C5—H5A	0.9500	O3W—H3WA	0.8500
C6—C7	1.519 (2)	O3W—H3WB	0.8501
N2—C9	1.324 (2)	O4W—H4WA	0.8501
N2—C8	1.353 (2)	O4W—H4WB	0.8523
			
C6—N1—C2	122.33 (15)	O4—C7—C6	113.36 (15)
C6—N1—H1N	121.0	C9—N2—C8	117.74 (14)
C2—N1—H1N	116.7	C11—N3—C8	120.82 (14)
C4—O3—H3O	111.5	C11—N3—H3N	120.2
O2—C1—O1	128.19 (15)	C8—N3—H3N	119.0
O2—C1—C2	116.07 (15)	C8—N4—H4NA	121.0
O1—C1—C2	115.72 (15)	C8—N4—H4NB	116.8
N1—C2—C3	119.94 (14)	H4NA—N4—H4NB	121.7
N1—C2—C1	116.36 (15)	N4—C8—N2	119.81 (14)
C3—C2—C1	123.68 (14)	N4—C8—N3	119.12 (14)
C2—C3—C4	119.80 (14)	N2—C8—N3	121.07 (15)
C2—C3—H3A	120.1	N2—C9—C10	123.61 (15)
C4—C3—H3A	120.1	N2—C9—H9A	118.2
O3—C4—C3	118.81 (14)	C10—C9—H9A	118.2
O3—C4—C5	122.94 (14)	C11—C10—C9	116.72 (16)
C3—C4—C5	118.25 (15)	C11—C10—H10A	121.6
C6—C5—C4	119.49 (14)	C9—C10—H10A	121.6
C6—C5—H5A	120.3	N3—C11—C10	119.98 (15)
C4—C5—H5A	120.3	N3—C11—H11A	120.0
N1—C6—C5	120.14 (15)	C10—C11—H11A	120.0
N1—C6—C7	116.19 (15)	H1WA—O1W—H1WB	107.7
C5—C6—C7	123.67 (14)	H2WA—O2W—H2WB	107.3
O5—C7—O4	128.37 (16)	H3WA—O3W—H3WB	107.4
O5—C7—C6	118.28 (16)	H4WA—O4W—H4WB	107.2
			
C6—N1—C2—C3	−1.8 (3)	C4—C5—C6—N1	0.0 (3)
C6—N1—C2—C1	176.57 (15)	C4—C5—C6—C7	−179.86 (16)
O2—C1—C2—N1	−177.73 (15)	N1—C6—C7—O5	175.00 (18)
O1—C1—C2—N1	0.9 (2)	C5—C6—C7—O5	−5.1 (3)
O2—C1—C2—C3	0.5 (2)	N1—C6—C7—O4	−5.0 (2)
O1—C1—C2—C3	179.14 (16)	C5—C6—C7—O4	174.86 (17)
N1—C2—C3—C4	0.2 (2)	C9—N2—C8—N4	178.06 (16)
C1—C2—C3—C4	−178.04 (15)	C9—N2—C8—N3	−2.6 (2)
C2—C3—C4—O3	−178.17 (15)	C11—N3—C8—N4	−178.99 (15)
C2—C3—C4—C5	1.4 (2)	C11—N3—C8—N2	1.7 (2)
O3—C4—C5—C6	178.07 (16)	C8—N2—C9—C10	1.2 (3)
C3—C4—C5—C6	−1.5 (2)	N2—C9—C10—C11	1.2 (3)
C2—N1—C6—C5	1.7 (3)	C8—N3—C11—C10	0.8 (2)
C2—N1—C6—C7	−178.45 (16)	C9—C10—C11—N3	−2.1 (2)

### Hydrogen-bond geometry (Å, °)

**Table:**

*D*—H···*A*	*D*—H	H···*A*	*D*···*A*	*D*—H···*A*
N4—H4NA···O2	0.91	1.90	2.795 (2)	172
N1—H1N···O1	0.93	2.26	2.6639 (19)	105
N1—H1N···O4	0.93	2.27	2.618 (2)	101
N4—H4NB···N2^i^	0.89	2.11	2.990 (2)	171
N3—H3N···O1	0.93	1.76	2.683 (2)	171
O3—H3O···O4^ii^	0.95	1.55	2.4910 (19)	171

Symmetry codes:  (i) −*x*+3/2, −*y*+1/2, −*z*; (ii) −*x*+5/2, *y*−1/2, −*z*+1/2.
